# Quality of life in women with early-stage and metastatic hormone receptor-positive, HER2-negative breast cancer receiving endocrine therapy

**DOI:** 10.1093/oncolo/oyae146

**Published:** 2024-06-21

**Authors:** David O’Reilly, Abdul Rehman Farooq, Paul Nevins Selvadurai, Laura Sheehan, Karen Molan, Bindu Krishnanivas, Valerie Mullen, David McMahon, Danial Hadi, Ahmed Ahmed, Maeve Jennings, Hailey Carroll, Sonya Chew, Bojan Macanovic, Ciara O’Hanlon Brown, Sinéad A Noonan, Seamus O Reilly, Roisin M Connolly, Caitriona Cahir, Catherine M Kelly

**Affiliations:** CUH/UCC Cancer Centre, Wilton, Cork, T12 EC8P, Ireland; Molecular Medicine, RCSI University of Medicine and Health Sciences, Beaumont Hospital, The Smurfit Building, Dublin, D09 YD60, Ireland; CUH/UCC Cancer Centre, Wilton, Cork, T12 EC8P, Ireland; Cancer Trials, Mater Misercordiae University Hospital, Dublin, D07 AX57, Ireland; CUH/UCC Cancer Centre, Wilton, Cork, T12 EC8P, Ireland; CUH/UCC Cancer Centre, Wilton, Cork, T12 EC8P, Ireland; Cancer Trials, Mater Misercordiae University Hospital, Dublin, D07 AX57, Ireland; Cancer Trials, Mater Misercordiae University Hospital, Dublin, D07 AX57, Ireland; CUH/UCC Cancer Centre, Wilton, Cork, T12 EC8P, Ireland; CUH/UCC Cancer Centre, Wilton, Cork, T12 EC8P, Ireland; Medical Oncology, University Hospital Kerry, Tralee, V92 NX94, Ireland; CUH/UCC Cancer Centre, Wilton, Cork, T12 EC8P, Ireland; Cancer Trials, Mater Misercordiae University Hospital, Dublin, D07 AX57, Ireland; Medical Oncology, Galway University Hospital, Galway, H91 YR71, Ireland; CUH/UCC Cancer Centre, Wilton, Cork, T12 EC8P, Ireland; HOPE Directorate, Trinity St. James’ Cancer Institute, Dublin, D08 NH71, Ireland; CUH/UCC Cancer Centre, Wilton, Cork, T12 EC8P, Ireland; Medical Oncology, University Hospital Kerry, Tralee, V92 NX94, Ireland; CUH/UCC Cancer Centre, Wilton, Cork, T12 EC8P, Ireland; CUH/UCC Cancer Centre, Wilton, Cork, T12 EC8P, Ireland; Data Science Centre, R CSI University of Medicine and Health Sciences, Dublin, D02 YH72, Ireland; Medical Oncology, Mater Private Hospital, Dublin, D07 WKW8, Ireland

**Keywords:** breast cancer, quality of life, patient reported outcomes

## Abstract

**Introduction:**

Early discontinuation of endocrine therapy (ET) is higher among patients with early breast cancer (EBC) compared to patients with metastatic hormone receptor-positive (HR+) breast cancer (MBC). In our clinical experience the reasons for this may include a significant burden of ET side effects impacting quality of life (QOL) in patients with EBC. We hypothesized that QOL is lower in patients with HR + EBC compared to patients with HR + MBC on ET.

**Methods:**

We conducted a cross-sectional observational study to assess QOL utilizing FACT-ES & EORTC QLQ C30 tools among patients with EBC and MBC receiving ET across 5 Irish hospitals.

**Results:**

A total of 417 patients were enrolled—EBC (79% *n* = 331) and MBC 21% (*n* = 86). Using the FACT-ES, we found no difference in overall QOL by stage (139.2 vs 141, *P*  = .33). Patients with HR + MBC had a lower symptom burden from ET compared to HR + EBC (61.4 vs 54, *P* < .01). In adjusted multivariate linear regression models, there was no difference in QOL for patients with EBC and MBC receiving ET.

**Conclusions:**

There was no significant difference in overall QOL for patients with EBC and MBC. However, patients with EBC experienced more endocrine symptoms. In adjusted multivariate linear regression models, the stage did not predict QOL. Our results suggest that endocrine symptoms are significant contributors to impaired QOL for patients with EBC but the role of other determinants of QOL (eg, stage) is less clear. Future work could include the development of stage-specific QOL tools and utilization of electronic patient-reported outcomes (ePROs) to identify and manage emergent toxicities.

## Teaser

Quality of life as measured by FACT-ES and EORTC QLQ C30 tools is similar for patients with early and metastatic hormone receptors-positive breast cancer receiving endocrine therapy. This finding could reflect the higher burden of endocrine symptoms experienced in early breast cancer and the lack of stage-specific tools that capture the unique QOL issues facing women with metastatic disease.

**Implications for practice**

This article suggests that the quality of life of those with early breast cancer as measured by FACT-ES & EORTC QLQ C30 tools is not be higher than women with advanced disease. This may be due to the burden of endocrine toxicity experienced by patients with early stage breast cancer and reflect a need for better tools to measure QOL in the metastatic setting. Further work is needed to tailor the quality-of-life tools to patients with both early and advanced breast cancer and to support women with early breast cancer in managing endocrine toxicity.

## Introduction

Most patients diagnosed with breast cancer (BC) have hormone receptor (HR+)-positive disease.^1^ Endocrine therapy (ET) improves survival in HR + early-stage BC (EBC) and metastatic BC (MBC). For patients with HR + EBC treatment ranges from tamoxifen or an aromatase inhibitor (AI) alone to regimens that include ovarian function suppression (OFS) if premenopausal, and a cyclin-D kinase inhibitor (CDK4/6) inhibitor if they have high-risk disease. For patients with endocrine-sensitive HR + MBC, first-line therapy predominantly includes an AI or selective estrogen receptor-degrader or modifier (SERM/SERD) ± a CDK4/6 inhibitor ± a bone modifying agent (BMA).^2-4^

Most patients diagnosed with HR + EBC survive their cancer and for those with HR + MBC contemporary clinical trials have demonstrated a median overall survival (OS) of 64 months.^5^ Despite these positive statistics, managing the toxicities of ET remains a challenge, particularly in the adjuvant setting.^6,7^ Discontinuation rates for adjuvant ET range from 32% to 73% at the end of 5 years of treatment.^8^ In clinical trials, higher rates of early discontinuation have also been reported in EBC HR + BC compared to trials using similar drugs in pts with HR + MBC. The CDK4/6 inhibitors are now standard of care for patients with high risk EBC and MBC in combination with ET. In two contemporary trials of the CDK4/6 inhibitor palbociclib plus ET versus ET alone, the adjuvant trial reported early discontinuation rates of 42% compared to 4% discontinuation in the metastatic trial of the same treatment.^9,10^ The high discontinuation rates observed in consistent with our clinical experience that women with EBC experience a significant burden of endocrine side effects, which appears to impact their quality of life (QOL).

Based on these data and our clinical experience, we hypothesized that patients with HR + MBC receiving ET would have a better QOL compared to those with HR + EBC due to the significant burden of ET-related symptoms experienced by patients with EBC.

## Methods

### Study population

We conducted a cross-sectional observational study. Women were identified from the hospital records of outpatient clinics in 5 cancer centers across Ireland from December 2020 to July 2022. Eligibility criteria included; being ≥ 18 years; a diagnosis of HR + EBC or HR + MBC; having received at least 2 weeks of ET (±CDK4/6 inhibitors, ±OFS); ability to read and understand English and provide informed consent. Women were invited to complete a questionnaire (themselves or in person with a research nurse) measuring QOL and a range of sociodemographic, clinical and psychosocial variables. Ethical approval was granted by all participating hospital research ethics boards. All participants provided informed consent to participate in the study.

### Outcome measure

#### QOL and endocrine symptoms

QOL was measured using the Functional Assessment of Cancer Therapy (FACT-ES) and EORTC QLQ-C30.^11,12^ Briefly, FACT-ES assesses 4 primary domains of QOL; physical, social and family, emotional, and functional well-being. It includes a 19-item endocrine subscale which measures endocrine symptoms and adverse effects of ET. The EORTC QLQ-C30 assesses global health status, and the 5 functional scales assess physical functioning, role functioning, emotional functioning, cognitive functioning, and social functioning.

For both the FACT-ES and the EORTC QLQ-C30, the individual domain/functional scales were calculated using the predefined scoring program where participants had to have answered at least half of the questions in a domain/functional scale to be included in the subscale score for that domain. A higher overall QOL score and higher individual domain/functional scale scores indicate better QOL and higher scores on symptom scales indicate higher symptom burden.

### Covariates

Sociodemographic covariates included; age, education level, and occupational status. Education level was categorized as having third-level education versus not. Occupational status was categorized as working (employed or looking after family/home) or not working (unemployed/retired/unable to work).

Clinical and treatment covariates extracted from medical records included; type and duration of ET, ovarian function suppression (OFS) (Yes/No), CDK4/6 inhibitors (Yes/No) and duration of therapy, receipt of chemotherapy (Yes/No), type and setting (adjuvant, metastatic or both), and time since last chemotherapy. Treatment was categorized into the following subgroups; ET only; ET and OFS only; ET and CDK4/6 only; ET and CDK4/6 and OS.

Psychosocial covariates included resilience, coping skills and social support. The Brief Resilience Scale (BRS) is a 6-item scale that assesses the perceived ability to bounce back or recover from stress.^13^ Scores are averaged and range from 1 (low resilience) to 5 (high resilience). The BRIEF COPE (Coping Orientation to Problems Experienced) is a 28-item scale designed to measure effective and ineffective ways to cope with a stressful life events.^14^ Two main coping strategies were measured; avoidant coping and approach coping. Social support was measured using the Modified Social Support Survey (MSSS-5), which provides an overall assessment of tangible support, emotional/informational support, affectionate support, and positive social interaction.^15^

### Sample size

The minimal important difference (MID) for interpreting group differences or changes in QOL over time for FACT-ES scales was estimated to be in the range of 3-8 points. To detect a difference of 7 points, we used a 3:1 study design and estimated that *N* = 142 patients with MBC and *N* = 426 patients with EBC would be sufficient based on 80% power and 5% significance level of showing a difference of 7 points as being statistically significant.

### Data analysis

Descriptive statistics including medians (interquartile range, IQR) and proportions, were calculated for QOL and treatment-related symptoms (FACT-ES, EORTC QLQ-C30) and all sociodemographic, clinical treatment, and psychosocial variables. Differences between these variables in EBC and MBC were examined using Chi-squared and Fisher’s exact tests for categorical variables and non-parametric Wilcoxon–Mann-Whitney tests for continuous variables.

The association between EBC and MBC and overall QOL (FACT-ES) (and for each individual domain and treatment-related symptoms) was assessed using multivariable linear regression analysis with adjustment for sociodemographic, clinical and treatment and psychosocial covariates using robust standard errors. We used robust standard errors controls for mild violation of the homoskedasticity and normality distribution assumption in linear regression. The data was analyzed using Stata Version 17.0 (StataCorp, College Station).

## Results

A total of 417 patients were recruited across the 5 hospital sites between December 2020 and July 2022; 331 (79%) had EBC and 86 (21%) MBC. Median age was significantly higher in patients with MBC at 61 (IQR; 53-73) compared to 57 years (IQR; 50-65) for EBC. Education status was similar between both groups. More patients with EBC were working compared to those with MBC (*P* < 0.01). Almost 40% (*n* = 126) of patients with EBC were taking tamoxifen compared to 13% (*n* = 11) with MBC. Use of OFS was higher in patients with MBC at 19.8% (*n* = 33) compared to 10% (*n* = 17) (*P* < .05) in EBC. CDK4/6 inhibitor use was significantly higher in pts with MBC (*n* = 55, 64%) compared to EBC (*n* = 6, 1%), *P* < .01). The median duration of ET was longer in patients with EBC at 27 months (IQR 13-48) compared to 19 months (IQR 8-35) for those with MBC (*P* < .01). However, the majority of patients in both groups had received over 12 months of ET (Table 1). There were higher rates of prior discontinuation for patients in the EBC (18%, 60/331) versus the MBC group (6%, 8/86).

**Table 1.** Socio-demographic, clinical, and treatment characteristics of women by EBC and MBC.

	EBC *N* = 331	MBC *N* = 86	*P*-value
Sociodemographic covariates			
Age (median, IQR)	57 (50, 65)	61 (50, 73)	.01^*^
*Education level*	*N* (%)	*N* (%)	
Primary and/or secondary	144 (44.9)	43 (52.4)	.22
Third level or post-graduate	177 (55.1)	39 (47.6)	
*Occupational status*	*N* (%)	*N* (%)	
Working	193 (59.8)	25 (30.5)	.01^*^
Not working	130 (40.2)	57 (69.5)	
Clinical and treatment-related covariates			
*Endocrine therapy*	*N* (%)	*N* (%)	
Tamoxifen	126 (38.4)	11 (13.3)	.01
Anastrazole/letrozole	175 (53.4)	37 (44.6)	
Exemesthane	23 (7.0)	6 (7.2)	
Fulvestrant	4 (1.2)	29 (34.9)	
Time on endocrine therapy (median months, IQR)	27 (13, 48)	19 (8, 35)	.01
*CDK4/6 inhibitor*	*N* (%)	*N* (%)	
Yes	6 (1.8)	55 (63.9)	.01
No	325 (98.2)	31 (36.1)	
Time on CDK4/6 inhibitor (median months, IQR)^a^	13.5 (10, 23)	15.5 (6, 25)	.86
*Ovarian suppression*	*N* (%)	*N*(%)	
Yes	33 (10.0)	17 (19.8)	.05
No	298 (90.0)	69 (80.2)	
*Chemotherapy*	*N* (%)	*N* (%)	
Yes	206 (62.2)	47 (54.7)	.20
No	125 (37.8)	39 (45.3)	
*Chemotherapy setting* ^a^	*N* (%)	*N* (%)	
Adjuvant	206 (100)	40 (85.1)	.01
Metastatic	0 (0)	7 (14.9)	
*Type of chemotherapy* ^a^	*N* (%)	*N* (%)	
ACT	128 (64.0)	28 (59.6)	.05
TC	33 (16.5)	4 (8.5)	
CMF	15 (7.5)	1 (2.1)	
Paclitaxel	16 (8.0)	7 (14.9)	
Other	8 (4.0)	7 (14.9%)	
Time since last chemotherapy (median months, IQR)^a^	45 (26, 65)	57 (35, 127)	.05
Psychosocial covariates	Median (IQR)	Median (IQR)	
Brief Resilience Scale	2.8 (2.7, 3.2)	3 (2.8, 3.2)	.05
Coping			
Avoidant	19 (15, 22)	18 (15, 22)	.36
Approach	31 (24, 36)	30 (23, 34)	.15
Modified social support survey (MSSS-6)	80 (50, 95)	85 (65, 100)	.08

^a^These measures apply only to those currently taking a CDK4/6 inhibitor or receiving chemotherapy.

Chi-square and Fishers tests for categorical data and Wilcoxon-Mann-Whitney tests for continuous data (*P* < .05). Missing *n*, *N* = 14 education level, *N* = 12 occupational status, *N* = 6 endocrine therapy, *N* = 6 type of chemotherapy, *N* = 4 time since last chemotherapy, *N* = 14 Brief Resilience scale, *N* = 4 MSSS-6.

Resilience, as measured by Brief Resilience Scale (scoring range 1-5), was significantly higher in patients with MBC (3, IQR 2.8 to 3.2) compared to EBC (2.8, IQR 2.7-3.2) *P* < .05. There were no statistically significant differences in coping strategies or social support between patients with EBC compared to MBC.

### Quality of life measures

There was no statistically significant difference in overall QOL between EBC (139.2 (IQR 113-156)) compared to MBC (141 (IQR 120.5-156.3)) by the FACT-ES tool. Endocrine symptoms were significantly higher in patients with EBC (54, IQR 42.2–64) compared to those with MBC (61.4, IQR 51.5-67), *P* < .01. Functional and emotional well-being was better in women with EBC compared to those with MBC (*P* < .05). There was no difference in social and physical well-being between the EBC and MBC (Table 2).

**Table 2.** Median (with IQRs) overall QOL, individual domain/functional scale scores and endocrine symptoms in patients with EBC and MBC.

FACT-ES	EBC (*N* = 331)		MBC (*N* = 86)		*P*-value
	*N*	Median (IQR)	*N*	Median (IQR)	
Overall (QOL)	324	139.2 (113 156)	84	141 (120.5,156.3)	.33
Physical well-being	328	23 (19,26)	86	23 (19,26)	.83
Social well-being	329	24 (19.8,26.8)	86	23.4 (18.0,26.8)	.87
Functional well-being	330	21 (16, 25)	85	19 (15, 23)	*<*.05
Emotional well-being	331	18 (14, 21)	86	17 (13, 20)	*<*.05
Endocrine symptoms	328	54 (42.2,64)	84	61.4 (51.4,67)	*<*.01
EORTC QLQ-C30					
Global health status	326	75.0 (58.3,83.3)	86	66.7 (50,83.3)	.12
*Functioning scales*					
Physical functioning	330	86.7 (66.7,93.3)	85	73.3 (53.3,86.7)	*<*.01
Role functioning	329	83.3 (66.7,100)	85	83.3 (50 100)	*<*.05
Emotional functioning	326	75 (58.3,91.7)	86	75 (66.7,91.7)	.27
Cognitive functioning	326	83.3 (50 100)]	86	83.3 (66.7,100)]	*<*.05
Social functioning	325	100 (66.7,100)	86	83.3 (50 100)	.07

^*^Wilcoxon-Mann-Whitney test: a higher overall QOL (EORTC QLQ-C30, FACT-ES), individual domain score or endocrine symptom score indicates a higher/better QOL (or lower symptom burden).

Abbreviations: IQR, inter-quartile range; QOL, quality of life;

For the EORTC QLQ-C30, we observed no difference in global health status between EBC and MBC at 75 (IQR 58.3-83.3) and 66.7 (IQR 50-83.3), respectively. EBC patients reported significantly higher physical functioning (*P* < .01), role functioning (*P* < .05), and cognitive functioning (P < .05) compared to those with MBC (Table 2).

### Multivariable analysis: associations between EBC and MBC and QOL and endocrine symptoms

Associations between EBC and MBC, FACT-ES, individual domains, and the endocrine subscale with adjustment for sociodemographic and clinical and treatment covariates are presented in Table 3. There were no statistically significant associations among the EBC and MBC and QOL (overall or the individual domains).

**Table 3.** Unadjusted and adjusted coefficients (SE) for FACT-ES, individual domains, and endocrine symptoms by breast cancer care setting (metastatic vs adjuvant), and sociodemographic, clinical, treatment, and psychosocial factors (*N* = 377).

	FACT-ES		Physical		Social		Functional		Emotional		Endocrine symptoms	
	Unadjusted β (SE)	Adjusted β (SE)	Unadjusted β (SE)	Adjusted β (SE)	Unadjusted β (SE)	Adjusted β (SE)	Unadjusted β (SE)	Adjusted β (SE)	Unadjusted β (SE)	Adjusted β (SE)	Unadjusted β (SE)	Adjusted β (SE)
*Breast cancer setting (vs EBC)*												
MBC	3.86 (3.34)	−1.54 (3.83)	−0.37 (0.66)	−0.68 (0.97)	0.08 (0.65)	−0.02 (0.84)	−1.15 (0.71)	−1.45 (1.07)	−1.05 (0.59)	−1.15 (0.83)	5.86 (1.66)^*^	1.79 (1.74)
*Sociodemographic covariates*												
Age	0.78 (0.12)^*^	0.73 (0.13)^*^	0.09 (0.03)^*^	0.09 (0.03)^*^	0.10 (0.02)^*^	0.08 (0.03)^*^	0.05 (0.03)	0.05 (0.03)	0.08 (0.02)^*^	0.08 (0.02)^*^	0.46 (0.06)^*^	0.42 (0.07)^*^
*Education (vs. primary and secondary level)*												
Third-level or postgraduate	−3.87 (2.76)	2.38 (2.52)	−0.73 (0.18)	−0.04 (0.54)	−1.25 (0.54)^*^	−0.52 (0.50)	−0.25 (0.59)	−0.23 (0.60)	0.08 (0.49)	0.71 (0.49)	−1.95 (1.39)	2.00 (1.34)
*Occupational status (vs. not working)*												
Working	6.11 (2.76)^*^	7.89 (2.52)^*^	2.34 (0.54)^*^	2.32 (0.54)^*^	0.07 (0.54)	0.01 (0.50)	2.34 (0.58)^*^	2.10 (0.60)^*^	0.92 (0.49)	0.73 (0.46)	0.10 (1.40)	2.56 (1.36)
Clinical and treatment covariates												
*Treatment (vs single agent ET or ET and ovarian sup pression)*												
ET and CDK46 or ET and ovarian suppression and CDK46	2.66 (3.84)	1.65 (4.65)	−0.21 (0.75)	0.48 (1.07)	−0.59 (0.75)	−1.07 (0.93)	−1.07 (0.82)	0.10 (1.19)	−1.29 (0.68)	−0.63 (0.96)	5.21 (1.93)^*^	2.64 (2.20)
Time on endocrine therapy	0.09 (0.05)	0.02 (0.04)	0.02 (0.01)	0.01 (0.01)	0.01 (0.01)	0.01 (0.01)	0.03 (0.01)^*^	0.02 (0.01)^*^	0.02 (0.01)	0.00 (0.01)	0.02 (0.03)	−0.02 (0.02)
*Chemotherapy (vs No)*												
Yes	−2.29 (2.77)	2.28 (2.42)	0.14 (0.55)	0.79 (0.54)	−1.04 (0.54)	−0.22 (0.49)	0.21 (0.59)	0.34 (0.58)	−0.28 (0.49)	0.26 (0.48)	−1.70 (1.39)	0.94 (1.32)
*Psychosocial covariates*												
Resilience	16.97 (2.81)^*^	8.00 (3.04)^*^	2.54 (0.57)^*^	1.20 (0.65)	3.23 (0.56)^*^	1.65 (0.80)^*^	3.45 (0.61)^*^	2.37 (0.76)^*^	2.49 (0.52)^*^	1.18 (0.57)^*^	5.28 (1.45)^*^	1.66 (1.57)
*Coping*												
Avoidant	−2.41 (0.23)^*^	−1.69 (0.29)^*^	−0.39 (0.05)^*^	−0.23 (0.07)^*^	−0.26 (0.05)	−0.15 (0.06)^*^	−0.27 (0.05)^*^	−0.21 (0.06)^*^	−0.42 (0.04)^*^	−0.42 (0.06)^*^	−1.10 (0.12)^*^	−0.69 (0.15)^*^
Approach	−0.57 (0.16)^*^	0.13 (0.17)	−0.13 (0.03)^*^	−0.03 (0.03)	0.02 (0.03)	0.10 (0.03)^*^	−0.01 (0.04)	0.06 (0.04)	−0.06 (0.03)^*^	0.07 (0.03)	−0.40 (0.08)^*^	−0.06 (0.09)
Social Support (MSSS−6)	1.96 (0.25)^*^	0.25 (0.05)^*^	0.29 (0.05)^*^	0.04 (0.01)^*^	0.53 (0.05)^*^	0.09 (0.01)^*^	0.33 (0.06)^*^	0.04 (0.01)^*^	0.24 (0.05)^*^	0.02 (0.01)^*^	0.57 (0.13)^*^	0.06 (0.03)

^*^ *P* < .05.

In terms of sociodemographic, clinical, and treatment covariates: increasing age was associated with a significantly higher overall, physical, social, and emotional QOL and a lower endocrine symptom burden. Working was associated with a higher overall, physical, and functional QOL. Increased time on ET was associated with a significantly higher functional QOL (*β* = 0.02 SE 0.01, *P* < .05) (Table 3).

Greater resilience (*β* = 8.00 SE 3.04, *P* < .01) was associated with a significantly higher overall, social, functional, and emotional QOL. The use of avoidant coping strategies was associated with a significantly reduced overall QOL (*β* = −1.69, SE 0.29, *P* < .01), physical, social, functional, and emotional QOL, and higher ET symptom burden. Increasing social support (*β* = 0.25 SE 0.05, *P* < .01) was associated with a significantly higher overall QOL and physical, social, functional, and emotional QOL (Table 3).

## Discussion

In this study, we found no difference in QOL between patients with EBC and MBC receiving ET for HR+, HER2 negative BC. Consistent with our hypothesis, we observed higher reported endocrine symptoms in patients with EBC compared to MBC. Our work supports the need for further support for patients on ET—such as better social and emotional support networks. Furthermore, our work supports the need for more refined QOL tools that take into account disease stage and the need for global, real-world quality of life studies. These approaches would allow us to comprehensively assess the most significant factors negatively impacting QOL for patients with BC, and to focus support on those key areas. It is plausible that given a diagnosis of an incurable cancer but one that can be managed long-term with ET is perceived differently from the same treatment and side effects given in a curative setting. In MBC (unlike in EBC), radiological imaging provides regular positive reinforcement that ET is working and this also might increase acceptance of side effects. This may also explain the substantial difference in treatment discontinuation between EBC and MBC clinical trials of similar ETs. It also supports a recent observation that patients with MBC wait until symptoms become moderate to severe before reporting them to their oncologist.^16^

Our objective was to determine if QOL (using FACT ES) would counterintuitively show poorer QOL and global health status in patients with EBC compared to MBC. The FACT-B and EORTC QLQ-C30 tools are among the most frequently used QOL measures in recent phase III clinical trials studying ET in EBC and MBC (Supplementary Table S1). Reference values for these tools report lower QOL scores for MBC compared to EBC.^17^ Studies comparing QOL between EBC and MBC mostly report lower QOL in MBC compared to EBC.^18,19^ This would appear logical as one group has an incurable cancer compared to a group were most will survive their cancer. Our findings differ from these studies. An explanation for our findings and a limitation of some prior studies comparing QOL in EBC compared to MBC is the inclusion of all BC subtypes despite each having distinct clinical behaviors and diverse treatment options. It is very plausible our results would be similar to other studies had we included patients receiving chemotherapy after ET.

A strength of our study is only including patients with MBC on ET. We recruited patients from 5 cancer centers with both an urban and rural demographic which allowed for representative sampling. A limitation of our study is not accruing our planned number of patients due to time limitations. However, we consider it unlikely that our overall study results would have changed with further accrual given that we recruited >70% of patients with no difference noted in QOL. Although we have fewer patients with MBC than EBC, this was expected in our study design and we anticipated a 3:1 ratio of EBC to MBC given the demographic expectations in our population. A further limitation of our study was that our patients were receiving varied ET (eg, tamoxifen, letrozole ± CDK4/6 inhibitiors) and also were receiving therapy for different durations of time. However, the vast majority of patients were receiving therapy for greater than 12 months.^16,20,21^

In our cohort patients with EBC were younger, more were working and their duration of time on endocrine therapy was longer compared to those with MBC. A greater proportion of patients with EBC were taking tamoxifen (40% vs 13%) which is associated with more vasomotor symptoms compared to AIs.^22^ It is plausible that coping with these side effects in addition to real or perceived personal and societal expectations about resuming “normal” life while also living with a fear of recurrence would negatively impact QOL. However, it is notable that in our cohort more patients with MBC were receiving OFS (20% vs 10%) and CDK4/6 inhibitors which are also associated with side effects and the median duration of ET was also long at 19 months in this group.

Alternatively, our findings may reflect a limitation in our understanding of what are the important factors impacting QOL at this time point for patients with endocrine-sensitive MBC. If we do understand them how can we measure them? For patients with endocrine-sensitive, HER2-negative MBC as health care professionals (HCPs) we expect the time on first and second-line ET as the best opportunity to be on an effective treatment with relatively few side effects, freedom from weekly hospital visits for blood testing and intravenous chemotherapy. It is plausible that we share these optimistic expectations with patients and perhaps influence their perception too and willingness to discuss QOL challenges.

Resilience, approach coping strategies and social support were all found to be associated with improved QOL. We found resilience was significantly higher in patients with MBC compared to those with EBC and no difference in coping strategies or social support between the groups. A systematic review has indicated that resilience may help women with breast cancer mitigate the impacts of cancer-related symptom distress and improve QOL.^23^ There is also evidence of a positive association between resilience and the various subscales of QOL.^24^ Further evidence indicates that women who adopt appropriate coping strategies may be able to reduce their psychosocial distress, increase their levels of resilience and thereby improve their QOL.^25^ While social support has been shown to enable women with breast cancer to process their trauma, facilitate coping and increase resilience.^26^ Further research needed to understand the relationship between resilience, coping styles, social support and QOL and how they may interact differently for patients with EBC versus those with MBC.^27,28^

Our findings could be interpreted as (1) supporting our clinical observations that for patients with MBC QOL while on ET although no better is no worse compared to patients with EBC or (2) QOL tools are not sensitive enough to extricate the important factors impacting QOL in patients with MBC and/or (3) patients with MBC on ET may be less likely to report side effects and symptoms of their disease.^16^ Our work supports further research into developing QOL tools that are specific to BC subtype, stage and treatment type. Further work could explore the use of ePROs to identify and actively manage emerging toxicities for patients with BC, particularly EBC. Feedback from BC pt groups and health care professionals regarding our observations will be important to refine the next steps in our research in this area.

## Supplementary material

Supplementary material is available at *The Oncologist* online.

oyae146_suppl_Supplementary_Table

## Data Availability

The data underlying this article cannot be shared publicly as we do not have ethical approval to do so.
