# Correction to: Quality of life in women with early-stage and metastatic hormone receptor-positive, HER2-negative breast cancer receiving endocrine therapy

**DOI:** 10.1093/oncolo/oyae355

**Published:** 2025-01-23

This is a correction to: David O’Reilly, Abdul Rehman Farooq, Paul Nevins Selvadurai, Laura Sheehan, Karen Molan, Bindu Krishnanivas, Valerie Mullen, David McMahon, Danial Hadi, Ahmed Ahmed, Maeve Jennings, Hailey Carroll, Sonya Chew, Bojan Macanovic, Ciara O’Hanlon Brown, Sinéad A Noonan, Seamus O Reilly, Roisin M Connolly, Caitriona Cahir, Catherine M Kelly, Quality of life in women with early-stage and metastatic hormone receptor-positive, HER2-negative breast cancer receiving endocrine therapy, The Oncologist, Volume 29, Issue 10, October 2024, Pages 842–849, https://doi.org/10.1093/oncolo/oyae146

In the originally published version of the manuscript, in the section Quality of Life Measures, the second sentence should have read: “Endocrine symptoms were significantly higher in patients with EBC (54, IQR 42.2–64) compared to those with MBC (61.4, IQR 51.5-67), P <.01.” instead of: “Endocrine symptoms were significantly higher in patients with EBC (61.4, IQR 51.5-67) compared to those with MBC (54, IQR 42.2–64), P <.01.”

The error does not alter the final conclusion or overall results of the paper.

The emendation has been made to the article online only.

