# Supplementary material for: Quality of life in women with early-stage and metastatic hormone receptor-positive, HER2-negative breast cancer receiving endocrine therapy
Source: Oncologist. 2024 Jun 21;29(10):842–9. doi: 10.1093/oncolo/oyae146 (PMC11448882; doi:10.1093/oncolo/oyae146)
Supplement: oyae146_suppl_Supplementary_Table [file oyae146_suppl_supplementary_table.docx]

**Supplementary Table 1**

**Summary of Tools**

| **Tool** | **Domains tested** |
| --- | --- |
| **FACT-ES** | Physical Well-being |
|  | Social Well-being |
|  | Emotional Well-being |
|  | Functional Well-being |
|  | Endocrine subscale |
| **EORTC** |  |
|  | Global Health Status |
|  | Physical Functioning |
|  | Role Functioning |
|  | Emotional Functioning |
|  | Cognitive Functioning |
|  | Social Functioning |
|  |  |
| **Social Support Survey** | Social Support |
|  |  |
| **Brief Cope** | Avoidant |
|  | Approach |
|  |  |
| **Brief Resilience Scale** | Resilience |
|  |  |
|  |  |
